# Remarks on Dr. Wright’s Theory of Cholera

**Published:** 1866-08

**Authors:** D. W. Flora

**Affiliations:** 157 Cottage Grove Ave.; Chicago


ARTICLE XXVII.

### REMARKS ON DR. WRIGHT'S THEORY OF CHOLERA.

By D. W. FLORA, M.D., of Chicago.

From the regularity in the times of the advent of cholera, and the universality and sameness of its effects upon the human being, Dr. WRIGHT concludes that the predisposing cause is not found upon, nor within, the earth, but that some other planet or celestial body, in crossing the earth's orbit, leaves in space some influence imperceptible to the senses, but baleful to the health and life of mankind.

The Doctor might have included the inferior animals as coming under this "baleful influence." Preceding and accompanying the cholera in the year 1832, in India, Russia, and Poland, not only the cattle, but camels, dogs, goats, and poultry died from a peculiar plague or pestilence, while the rooks, daws, and migratory birds, perceiving this "baleful influence," took their untimely departure. In the department of the Seine, in France, the carp in the ponds died in great numbers during the cholera season. At each subsequent visitation of this scourge of mankind, the same phenomena have occurred, as witness the *rinderpest* in Russia, England, and other countries.

It is hardly necessary to mention the "miasmatic," the "animalcular," the "cholera cell," and the "cholera dust" theories. They are all contradicted by well-established facts. The facts and arguments in favor of contagion and against it are pretty equally balanced.

Dr. WRIGHT'S theory of celestial origin excludes all telluric influences as predisposing causes. In fact, I am disposed to regard them as merely effects or, at most, modifying causes. Dr. BARTHOLOW considers the special telluric influences favoring the rise, development, and spread of cholera to be, long-continued dryness of the atmosphere; a high temperature; a close and still atmosphere, with increased pressure, as indicated by the barometer; and *an almost total absence of ozone.* This last phenomenon I look upon as most important in directing us to the true cause of cholera; for, whether ozone be regarded as an independent element, or merely electrified oxygen, its presence in the atmosphere depends, in either case, upon electrical disturbance, and its absence indicates a corresponding want of electrical action.

So pleased am I with Dr. WRIGHT'S theory, that I am willing even to accompany him to the celestial bodies, to learn more of it. But the Doctor will not admit that this sidereal, solar, lunar, or cometary influence which produces cholera is appreciable by our dull senses, or any aids which we may bring to bear in our search for it.

There is one celestial influence which is known to be exerted on this earth, namely, the magnetic attraction of the sun and moon. This is demonstrated by the mariner's needle. Why may not the planets and other heavenly bodies exercise a similar influence? The Doctor says, most truthfully, that the sensible seat of cholera is in the lining membrane of the stomach and bowels, which is the same as saying that cholera attacks and destroys the nutritive or vegetative system of man, which is sometimes called the system of organic life. Little is said of the influence which electricity and magnetism exert upon this function which in animals and vegetables is the same, namely, absorption and assimilation. Some of my readers may remember the experiments made some years ago by applying galvanic currents to germinating and growing plants, by which these processes were greatly accelerated. Man is sometimes spoken of as though he was comparatively independent of certain earthly or telluric influences, the earth being considered merely as a "house" or "tabernacle." Nothing can be further from the truth. Man, with regard to this vegetative or organic life, is as much dependent upon the earth and surrounding elements as the plant upon its surface. His perfect organism (exceeding all others) is not life. It is passive—furnishing a condition of life merely. It is only by the action of such agencies as oxygen, hydrogen, light, heat, electricity, magnetism, etc., upon his body and the reaction of the body upon the same, which give us the phenomenon which we call life. Derange the relations which his organism bears to one of these agencies or elements and the functions of the body are proportionately deranged. Withdraw or destroy one or more of these external agents, and death of the organism is the result.

This nutritive system is spread out over a great surface, resembling the leaves of the plant, for it must be borne in mind that the leaves are more than lungs, they are digestive organs. How admirably this great surface is adapted to the electrical action or function which is always on the surface.

The Doctor, in speaking of the progress of an attack of cholera, says:—"That the natural attraction of the capillary system for the blood must not only be suspended, but instead of the constant affinity existing between the blood and the solids, resulting in health, in the molecular changes of reproduction and decay, cholera, either through morbid impressions upon the ultimate nervous fibrillæ, or through changes in the blood itself, or both together, induces a state of *positive* and *energetic repulsion* between the solids and liquids throughout the entire structure." What is the cause of this repulsion? What is the constant "affinity" mentioned as existing between the blood and the solids but magnetic attraction, and what is the "energetic repulsion" but magnetic or electrical? The body, as a whole, is a magnet; the blood globules, by reason of their iron, are magnetic also. In this mutual repulsion between fluids and solids, the bowels are entirely passive. This, Dr. WRIGHT recognizes, when he says:—"The fluids reverse violently their natural course of progression; not by reason of attraction, or disease, or any special condition of the bowels, but by *repulsion* from the general capillary, or peripheral, or ulterior tissues."

In speaking of cramps, and their cause, the Doctor remarks, "that although the respiration may be sufficient, it is plain to see that the vital changes in the blood are not perfectly performed." This is attributed to the fact "that the lungs suffer from the great drainage, in common with the rest of the system." Is not the office of the lungs, in a great degree, passive, as shown in artificial respiration, where they act more like a pair of bellows. The reason assigned above for the imperfect aëration of the blood cannot be the true one, so long as "inspiration and expiration are sufficient." May not this imperfect aëration, and also this repulsion between the blood and solids be due to *want of a proper amount of electricity in the inspired oxygen?*

May not this want of electricity, in the lung capillaries and surface capillaries of the body, be the cause of the inertia in the vascular system? The relation of positive and negative, as existing between the blood and these capillary systems, is probably changed, and hence the "energetic repulsion." Dr. WRIGHT'S pathology and treatment are eminently sound and rational, and he remarks, in view of the relative influence of fear as a depressing cause, and regimen as conservative to life: "It is a principle beyond cavil, that there is a certain amount of nervous energy natural to each personal organization developed in health within certain periods, no more, no less." This answers the question, why all persons do not have cholera when the cause is so universal. It is the want of this "nervous energy" which predisposes to cholera, as the presence of it in a high degree fortifies the possessor against the cholera influence. In regard to the electrical phenomena attending cholera, my personal experience is limited, as well as my opportunities of ascertaining the results of other men's observations.

During the year 1850, many of the towns and villages in Ohio were decimated. In a little village of 300 inhabitants, in which I resided at the time, there were 30 deaths. The weather was dry and not excessively warm. But one thunderstorm occurred during the cholera season, and it was followed by a marked abatement of the disease, so much so that fugitives returned and all congratulated themselves that the scourge had taken its departure. But we were doomed to disappointment. In a week or two it broke out with equal violence, and did not stop short of decimation. The absence of electrical phenomena was found to coincide with the absence of *ozone* in this locality. This has been the observation of several of my medical acquaintances, but I cannot claim from my own knowledge that it was a general condition.

A few words as to treatment, and I shall have done. Dr. Wright and some others which I have read, seem to be opposed to friction. Now, friction, with a view to arrest the cramps, can do no good, and may, as they claim, do harm by depleting the already collapsed vascular system. But gentle friction, surface rubbing with a hair glove or the dry hand to excite electrical action, cannot fail to do good. Nay, galvanism is strongly indicated, and when it is remembered that by it the temperature of a paralyzed limb has been raised 7° of Fah. it promises, if judiciously and scientifically applied, beneficial results. There is one fact to which I will briefly refer and then close.

All authorities which I have read recently, condemn the use of moisture and moist applications to the body. They all unite in saying that it favors *collapse.* This I can readily believe, for moisture is a bad conductor of electricity, and the poor patient by its application is cut off from even such an amount as might be generated by contact with the dry bedclothing and the atmosphere, hence, it is proper to use dry heat, dry mustard, and dry ice to the spine, as recommended by Dr. CHAPMAN, in his work on cholera.

*157 Cottage Grove Ave., June 19, 1866.*

